# Emergence of co-resistance to imipenem/relebactam and ceftazidime/avibactam in clinical *Klebsiella pneumoniae* ST11 clone due to KPC-2 N132S and CTX-M-65 S130G/P167S substitutions

**DOI:** 10.1128/aac.00891-25

**Published:** 2025-11-04

**Authors:** Yiqi Fu, Yuchao Zhang, Jun Zhao, Jiahong Xie, Haishen Kong, Tingting Yang, Wei Chen, Min Xu

**Affiliations:** 1 Department of Respiratory Diseases, The First Affiliated Hospital, Zhejiang University School of Medicine 26441, Hangzhou, China; 2 State Key Laboratory for Diagnosis and Treatment of Infectious Diseases, Collaborative Innovation Center for Diagnosis and Treatment of Infectious Diseases, The First Affiliated Hospital, Zhejiang University School of Medicine 26441, Hangzhou, China; 3 Department of Respiration, Zhejiang Medical & Health Group Hangzhou Hospital 713496, Hangzhou, China; 4 Department of Food Science and Nutrition, College of Biosystems Engineering and Food Science, Zhejiang University 366093 , Hangzhou, China; 5 Department of Laboratory Medicine, The First Affiliated Hospital, Zhejiang University School of Medicine 26441, Hangzhou, China; University of Fribourg, Fribourg, Switzerland

**Keywords:** imipenem/relebactam, ceftazidime/avibactam, *Klebsiella pneumoniae*, KPC, CTX-M

## Abstract

Two episodes of imipenem/relebactam and ceftazidime/avibactam co-resistant *Klebsiella pneumoniae* infections were identified in a Chinese hospital with an 8-month interval. All strains belonged to the ST11-KL64 clone, with pairwise SNP distances ranging from 1 to 9, indicating clonal spread. Plasmid-borne *bla*_KPC-157_ (KPC-2 N132S substitution) and *bla*_CTX-M-249_ (CTX-M-65 S130G/P167S substitutions) conferred resistance to imipenem/relebactam and ceftazidime/avibactam, respectively. The emergence of these novel resistance determinants in the high-risk clone underscores the need for enhanced surveillance.

## INTRODUCTION

Carbapenem-resistant *Klebsiella pneumoniae* (CRKP) infections have emerged worldwide, often associated with high mortality due to limited therapeutic options (1). *K. pneumoniae* carbapenemase (KPC) represents the most frequently encountered resistance mechanism in many countries (2). Although ceftazidime/avibactam (CAZ/AVI) initially showed promise against KPC-Kp, resistance is now rising, predominantly attributed to substitutions in KPC-2/KPC-3 (3). Mutations in other β-lactamases, including VEB (4), CTX-M (5, 6), CMY (7–9), and chromosomal AmpC (10), as well as overproduction of KPC (11), have been infrequently reported to confer resistance to CAZ/AVI.

Relebactam (REL), similar to AVI, has a diazabicyclooctane core but differs by equipping with a piperidine ring at the carbamoyl group, which enhances resistance to drug extrusion (12). Imipenem (IMI)/REL has shown activity against even CAZ/AVI-resistant KPC-Kp (13), and reports of clinical IMI/REL resistance due to KPC mutation are limited (14). Here, we described two cases caused by CAZ/AVI and IMI/REL co-resistant KPC-Kp and investigated the molecular basis of this resistance.

Patient 1, a 69-year-old male, was admitted in July 2023 due to neutropenia and septic shock following chemotherapy. Three CRKP isolates (XM610, XM755, and XM756) were isolated from blood, sputum, and stool cultures, respectively. Patient 2, a 79-year-old male, was admitted in February 2024 due to a surgical site abscess after vertebral abscess surgery in December 2023 in the same hospital. Pus culture yielded a CRKP isolate named XM991, and sputum culture returned two CRKP isolates (XM985 and XM997). The detailed antimicrobial treatment and timeline for the two patients are shown in Fig. S1.

All six isolates exhibited co-resistance to CAZ/AVI (64/4 or >64/4 mg/L) and IMI/REL (32/4 mg/L) (Table 1). Carbapenemase detection using NG-Test CARBA 5 (NG-Biotech, France) showed only positive for KPC. The inconsistency between phenotype and genotype prompted an investigation into potential mechanisms of resistance. Genomic DNA of the six isolates was extracted and sequenced by the Illumina platform with a PE150 strategy. For XM610 and XM991, long-read Nanopore sequencing was also applied using the PromethION platform. A detailed methodology for sequencing, assembly, annotation, and bioinformatic analysis is provided in the supplementary material.

**TABLE 1:** MICs and β-lactamase genes of the strains described in this study^*a*^

Strains	Description	β-Lactamase genes	MIC (mg/L)													
			IPM	IMI/REL	MEM	MER/VAB	CAZ	CAZ/AVI	CTX	FEP	TZP	ATM	ATM/AVI	FDC	TGC	PB
*K. pneumoniae*																
XM610	Clinical *K. pneumoniae* isolate	*bla*_KPC-157_, *bla*_CTX-M-65_, *bla*_CTX-M-249_, *bla*_TEM-1B_, *bla*_SHV-12_	32	32/4	> 32	8/8	> 64	> 64/4	> 32	> 32	> 256/4	> 256	2/4	2	2	0.5
XM991	Clinical *K. pneumoniae* isolate	*bla*_KPC-157_, *bla*_CTX-M-65_, *bla*_CTX-M-249_, *bla*_TEM-1B_, *bla*_SHV-12_	32	32/4	> 32	8/8	> 64	64/4	> 32	> 32	> 256/4	> 256	2/4	2	1	1
XM876	Clinical carbapenem-susceptible ST11-KL64 *K. pneumoniae* isolate	*bla*_TEM-1B_	≤ 0.25	≤ 0.25/4	≤ 0.25	≤0.5/8	1	≤ 0.5/4	≤ 0.25	0.5	128/4	0.5	≤ 0.25/4	≤ 0.25	0.5	0.5
XM876-pXM610-2	Transformant of plasmid pXM610-2	*bla*_KPC-157_, *bla*_CTX-M-65_, *bla*_TEM-1B_, *bla*_SHV-12_	16	8/4	> 32	4/8	> 64	2/4	> 32	> 32	> 256/4	> 256	1/4	1	0.5	0.5
XM876-pXM610-4	Transformant of plasmid pXM610-4	*bla*_CTX-M-249_	≤ 0.25	≤ 0.25/4	≤ 0.25	≤ 0.5/8	> 64	> 64/4	4	16	256/4	2	0.5/4	≤ 0.25	0.5	0.5
*E. coli*																
EC600-pXM610-2	Conjugant-containing plasmid pXM610-2	*bla*_KPC-157_, *bla*_CTX-M-65_, *bla*_TEM-1B_, *bla*_SHV-12_	1	1/4	1	≤ 0.5/8	> 64	≤ 0.5/4	> 32	4	8/4	> 256	≤ 0.25/4	≤ 0.25	≤ 0.25	0.5
DH5α-pXM610-2	Transformant of plasmid pXM610-2	*bla*_KPC-157_, *bla*_CTX-M-65_, *bla*_TEM-1B_, *bla*_SHV-12_	2	1/4	2	≤ 0.5/8	> 64	≤ 0.5/4	> 32	4	8/4	> 256	≤ 0.25/4	1	≤ 0.25	0.5
DH5α-pXM610-4	Transformant of plasmid pXM610-4	*bla*_CTX-M-249_	≤ 0.25	≤ 0.25/4	≤ 0.25	≤ 0.5/8	> 64	> 64/4	0.5	2	32/4	0.125	≤ 0.25/4	0.5	≤ 0.25	0.5
DH5α-pET28a_KPC-157	Transformant of recombinant plasmid pET28a_KPC-157	*bla*_KPC-157_	4	4/4	4	≤ 0.5/8	2	≤ 0.5/4	≤ 0.25	≤ 0.25	128/4	2	≤ 0.25/4	≤ 0.25	≤ 0.25	0.5
DH5α-pET28a_KPC-2	Transformant of recombinant plasmid pET28a_KPC-2	*bla*_KPC-2_	8	≤ 0.25/4	8	≤ 0.5/8	64	≤ 0.5/4	> 32	16	> 256/4	> 256	≤ 0.25/4	≤ 0.25	≤0 .25	0.5
DH5α-pET28a_CTX-M-249	Transformant of recombinant plasmid pET28a_CTX-M-249	*bla*_CTX-M-249_	≤ 0.25	≤ 0.25/4	≤ 0.25	≤ 0.5/8	> 64	64/4	1	1	64/4	1	≤ 0.25/4	≤ 0.25	≤ 0.25	0.5
DH5α-pET28a_CTX-M-65	Transformant of recombinant plasmid pET28a_CTX-M-65	*bla*_CTX-M-65_	≤ 0.25	≤ 0.25/4	≤ 0.25	≤ 0.5/8	8	≤ 0.5/4	> 32	2	≤ 2/4	> 256	≤ 0.25/4	≤ 0.25	≤ 0.25	0.5
DH5α	Recipient of electroporation	None	≤ 0.25	≤ 0.25/4	≤ 0.25	≤ 0.5/8	≤ 0.5	≤ 0.5/4	≤ 0.25	≤0.25	≤ 2/4	≤ 0.064	≤ 0.25/4	≤ 0.25	≤ 0.25	0.5
EC600	Recipient of conjugation	None	≤ 0.25	≤ 0.25/4	≤ 0.25	≤ 0.5/8	≤ 0.5	≤ 0.5/4	≤ 0.25	≤0.25	≤ 2/4	≤ 0.064	≤ 0.25/4	≤ 0.25	≤ 0.25	0.5

^*a*^ IPM, imipenem; IMI/REL, imipenem/relebactam; MEM, meropenem; MER/VAB, meropenem/vaborbactam; CTX, cefotaxime; CAZ, ceftazidime; CAZ/AVI, ceftazidime/avibactam; FEP, cefepime; TZP, piperacillin/tazobactam; ATM, aztreonam; ATM/AVI, aztreonam/avibactam; FDC, cefiderocol; TGC, tigecycline; PB, polymyxin B.

All six isolates belonged to the ST11-KL64 clone, with only a few single-nucleotide polymorphisms (SNPs) ranging from 1 to 9 bps among them (Fig. S2), indicating potential clonal spread during the 8-month period. All isolates exhibited an identical resistance gene profile, highlighted by two rare β-lactamase genes, *bla*_KPC-157_ and *bla*_CTX-M-249_ (Table S1). KPC-157 was identical to KPC-2 except for an N132S substitution, and CTX-M-249 differed from CTX-M-65 by two non-synonymous amino acid substitutions, S130G and P167S (Ambler numbering scheme). XM610 was serially passaged for 30 days in antibiotic-free MH agar. Both *bla*_KPC-157_ and *bla*_CTX-M-249_ remained unchanged, demonstrating high genetic stability of the two mutations in the absence of selective pressure.

*bla*_KPC-157_ was located on a 141,448 bp IncFII-like replicon type (81.32% identity with IncFII) plasmid, pXM610-2 (Table S1). Electroporation of pXM610-2 elevated the IMI/REL MIC in *Escherichia coli* DH5α at least fourfold (from ≤0.25/4 to 1/4 mg/L), whereas it produced at least 32-fold increase in *K. pneumoniae* XM876 (from ≤0.25/4 to 8/4 mg/L) (Table 1). XM876 is a carbapenem-susceptible ST11 clinical strain from our hospital that possesses an identical porin gene profile to XM610 (Table S1). The results indicate that the loss of porins potentiates the resistance conferred by pXM610-2. Transformation of DH5α with recombinant plasmid pET-28a*-bla*_KPC-157_ (containing *bla*_KPC-157_ and its native promoter, see Supplemental Material) markedly increased the IMI/REL MIC (from ≤0.25/4 to 4/4 mg/L) (Table 1). Conjugation experiments showed that pXM610-2 could be successfully transferred from XM610 to rifampin-resistant *E. coli* EC600 at a frequency of 1 × 10^−6^.

*bla*_CTX-M-249_ was the sole resistance gene identified on pXM610-4, an 88,638 bp hybrid IncFII:IncR plasmid (Table S1). Electroporation with pXM610-4 conferred CAZ/AVI resistance in DH5α and XM876 (MICs from ≤0.5/4 to >64/4 mg/L) (Table 1). The DH5α transformant of recombinant plasmid pET-28a-*bla*_CTX-M-249_ (containing *bla*_CTX-M-249_ and its native promoter, see Supplemental Material) exhibited resistance to CAZ/AVI (Table 1). Conjugation attempts to transfer pXM610-4 into EC600 failed.

**Fig 1:**
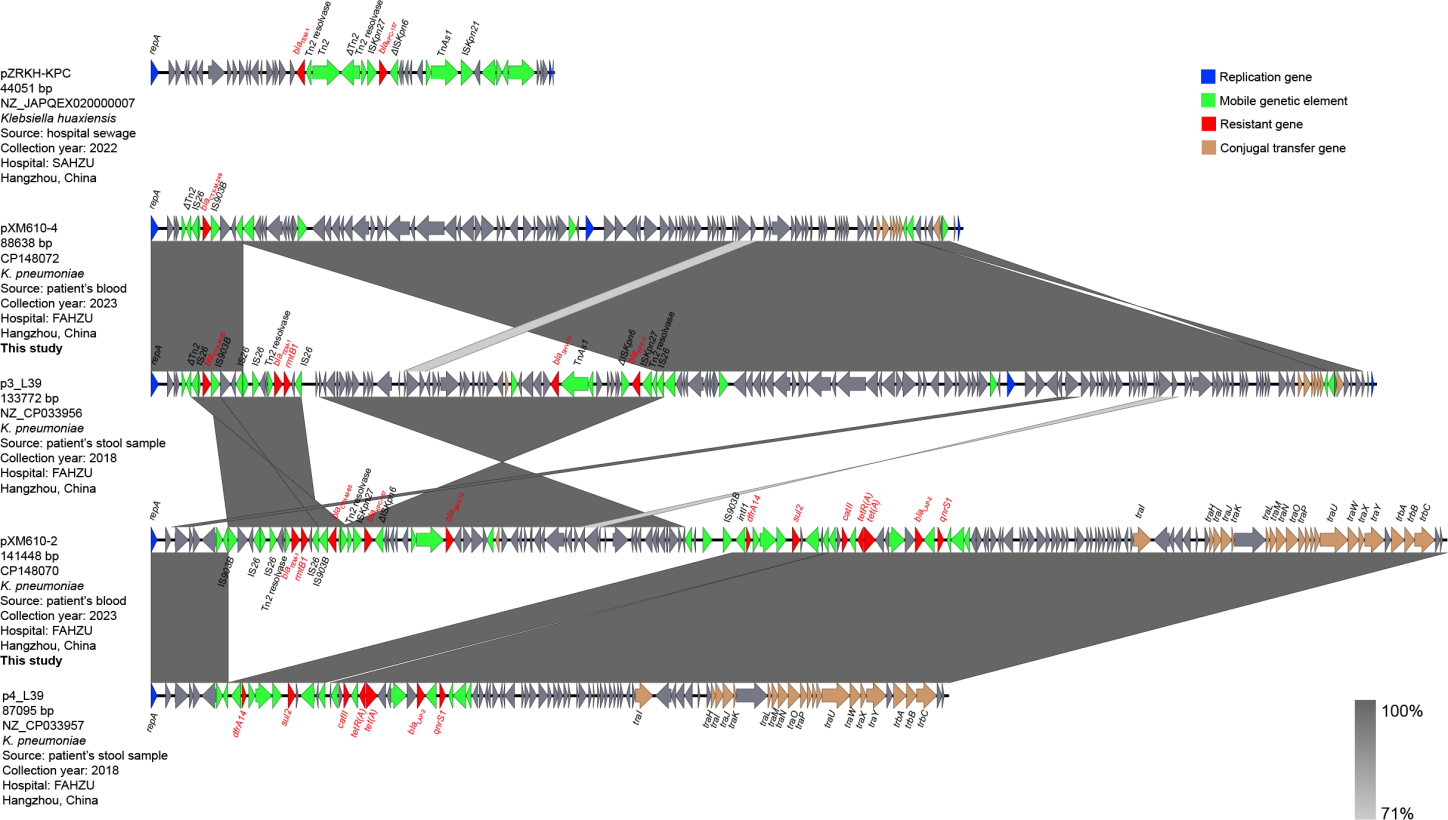
Structures of *bla*_KPC-157_-harboring plasmid pXM610-2 and *bla*_CTX-M-249_-harboring plasmid pXM610-4 and comparison with similar plasmids.

Plasmids pXM610-2 and pXM610-4 showed structural similarities to the plasmids p3_L39 and p4_L39 of a CRKP strain (L39_2) isolated from the same hospital in 2018 (GenBank accession No. NZ_CP033956 and NZ_CP033957) (Fig. 1). We hypothesize that pXM610-4 originated from p3_L39 after excision of the *bla*_KPC_-containing fragment, whereas pXM610-2 emerged when the conjugative plasmid p4_L39 acquired both the *bla*_KPC_ and *bla*_CTX-M-65_-containing fragments from p3_L39. This genetic event relocated *bla*_KPC_ into a transmissible plasmid, thereby equipping it with the capacity for horizontal dissemination.

A GenBank survey revealed that as early as 2022, a *Klebsiella huaxiensis* strain (ZR-9) (GenBank accession No. NZ_JAPQEX020000007) isolated from the wastewater of the Second Affiliated Hospital of Zhejiang University School of Medicine, located only approximately 900 m from our hospital, already carried plasmid-borne *bla*_KPC-157_ flanked by the identical Tn*2*-IS*Kpn27-bla*_KPC-157_-ΔIS*Kpn6* arrangement observed in pXM610-2 (Fig. 1). Meanwhile, *bla*_CTX-M-249_ was present in a *K. pneumoniae* strain KP-ICU-3 deposited in July 2021 (GenBank accession No. MZ379782) by investigators at Sir Run Run Shaw Hospital, Zhejiang University School of Medicine, which is approximately 3 km from our hospital. Together, these findings indicate that both genes may have been silently circulating in the local region and environmental reservoirs, potentially contributing to the spread of *bla*_KPC-157_.

Here, we describe the first clinical ST11-KL64 CRKP strains simultaneously producing two exceptionally rare β-lactamases, KPC-157 and CTX-M-249, conferring high-level resistance to both IMI/REL and CAZ/AVI. To date, resistance to CAZ/AVI associated with CTX-M substitutions has scarcely been reported (5, 6). This study provides the first clinical documentation of CTX-M-249 in *K. pneumoniae* with no prior exposure to CAZ/AVI. CTX-M-249 harbors dual substitutions (P167S and S130G) that act synergistically to confer CAZ/AVI resistance. P167S enlarges the active site via Ω-loop conformational change, enhancing ceftazidime hydrolysis (15–17), while S130G disrupts avibactam binding and recyclization (18, 19). This dual-mutation mechanism aligns with prior studies showing that a single substitution in CTX-M is insufficient for CAZ/AVI resistance, and only combinations like S130G/L169Q or P167S/T261I yield high MICs (5, 6).

In KPC-Kp, increased *bla*_KPC_ copy number, KPC-3 L167R substitution, and porin defects are associated with IMI/REL resistance (11, 13, 20, 21). KPC-157, which confers relebactam resistance, was initially selected *in vitro* under IMI/REL exposure (14). The N132S substitution prevents interaction with relebactam, causing a conformational shift in the binding of relebactam to KPC-157 and leading to the loss of inhibitory activity (14). Our study presents the first clinical isolates of *K. pneumoniae* producing KPC-157, marking a significant shift from laboratory observations to real-world clinical scenarios.

Notably, aztreonam/avibactam still retained potent activity against the *K. pneumoniae* in this study (MIC ≤2/4 mg/L) (Table 1), indicating that this combination remains a viable therapeutic option for infections caused by KPC-157 and CTX-M-249 co-producing strains.

Lastly, ST11-KL64 is now recognized as the most formidable CRKP sub-clone in China: it unites extreme multidrug resistance with exceptional transmissibility and is intimately linked to high mortality (22). The convergence of *bla*_CTX-M-249_ and *bla*_KPC-157_ within this background intensifies the threat, producing a novel sub-clone with simultaneous resistance to last-line agents CAZ/AVI and IMI/REL, which necessitates urgent and enhanced surveillance to curtail further spread.

## Data Availability

The complete sequences of the chromosomes and plasmids of XM610 and XM991 have been deposited in the GenBank database under accession numbers from CP148068 to CP148073 and CP152054 to CP152059, respectively.

## SUPPLEMENTAL MATERIAL

Supplemental material aac.00891-25-s0001.docx Fig. S1 and S2; Table S1.

ASM does not own the copyrights to Supplemental Material that may be linked to, or accessed through, an article. The authors have granted ASM a non-exclusive, world-wide license to publish the Supplemental Material files. Please contact the corresponding author directly for reuse.
