# Supplementary material for: Emergence of co-resistance to imipenem/relebactam and ceftazidime/avibactam in clinical Klebsiella pneumoniae ST11 clone due to KPC-2 N132S and CTX-M-65 S130G/P167S substitutions
Source: Antimicrob Agents Chemother. 2025 Nov 4;69(12):e00891-25. doi: 10.1128/aac.00891-25 (PMC12691654; doi:10.1128/aac.00891-25)
Supplement: Supplemental material — Fig. S1 and S2; Table S1. [file aac.00891-25-s0001.docx]

**Supplementary Data for:**

**Emergence of co-resistance to imipenem/relebactam and ceftazidime/avibactam in clinical *Klebsiella pneumoniae* ST11 clone due to KPC-2 N132S and CTX-M-65 S130G/P167S substitutions**

Yiqi Fu^1^, Yuchao Zhang^2^, Jun Zhao^3^, Jiahong Xie^4^, Haishen Kong^2^, Tingting Yang^5^, Wei Chen^4#^, Min Xu^5#^

^1^Department of Respiratory Diseases, The First Affiliated Hospital, Zhejiang University School of Medicine, Hangzhou, China

^2^State Key Laboratory for Diagnosis and Treatment of Infectious Diseases, Collaborative Innovation Center for Diagnosis and Treatment of Infectious Diseases, The First Affiliated Hospital, Zhejiang University School of Medicine, Hangzhou, China

^3^Department of Respiration, Zhejiang Medical & Health Group Hangzhou Hospital, Hangzhou, China

^4^Department of Food Science and Nutrition, College of Biosystems Engineering and Food Science, Zhejiang University, Hangzhou, China

^5^Department of Laboratory Medicine, The First Affiliated Hospital, Zhejiang University School of Medicine, Hangzhou, China

^#^Addresses for correspondence:

Min Xu, Department of Laboratory Medicine, The First Affiliated Hospital, Zhejiang University School of Medicine. No 79, Qingchun Road, Hangzhou 310003, China. Email: xu_min@zju.edu.cn. Telephone: +86 571 87236381, Fax: +86 571 87236381.

Wei Chen, Department of Food Science and Nutrition, College of Biosystems Engineering and Food Science, Zhejiang University. No 866, Yuhangtang Road, Hangzhou 310058, China. Email: zjuchenwei@zju.edu.cn. Telephone: +86 571 87076150, Fax: +86 571 88982191.

**Antimicrobial susceptibility testing**

Minimum inhibitory concentrations (MICs) of imipenem, imipenem/relebactam, meropenem, meropenem/vaborbactam, cefotaxime, ceftazidime, ceftazidime/avibactam, cefepime, piperacillin/tazobactam, aztreonam/avibactam, cefiderocol, tigecycline and polymyxin B were conducted by broth microdilution. All the antimicrobial powders were obtained from MedChem Express (New Jersey, USA). For tazobactam, avibactam and relebactam, a fixed concentration of 4 mg/L were used, whereas vaborbactam was tested at 8 mg/L. Iron-depleted cation-adjusted Mueller-Hinton broth was used for cefiderocol assay. MICs of aztreonam were determined using E-test strips. The results were interpreted according to the CLSI 2025 standard (1) except for tigecycline (FDA, 2019 breakpoints) and aztreonam/avibactam (EUCAST, 2025 standard) (2).

**Plasmid transformation and conjugation**

To better mimic the genetic background, we used *Klebsiella pneumoniae* XM876 as the recipient in electroporation experiments alongside the conventional *Escherichia coli* DH5α. *K. pneumoniae* XM876 is a carbapenem and ceftazidime susceptible ST11 strain isolated from the same hospital in November 2023. XM876 carries only one acquired β-lactamase gene *bla*_TEM-1B_, and notably, possesses the same porin gene profiles as XM610 (Table S1). The electrocompetent cells were prepared by a rapid microcentrifuge-based method (3). The transformants were selected on Mueller-Hinton agar (MHA, Oxoid, UK) plates with 8 mg/L ceftazidime.

For the conjugation assay, rifampicin-resistant *E. coli* EC600 was used as the recipient, and conjugants were selected on MHA plates containing 200 mg/L rifampicin and 8 mg/L ceftazidime. The limit of detection for conjugation from *K. pneumoniae* XM610 to rifampin-resistant *E. coli* EC600 was 6×10^-8^.

**Cloning experiments**

Gene fragments of *bla*_KPC-157_, *bla*_CTX-M-65_, *bla*_CTX-M-249_, and *bla*_KPC-2_, along with 500 bp upstream and 100 bp downstream sequences were amplified for cloning experiment. Identical flanking sequences allowed the same primers to amplify *bla*_KPC-157_/*bla*_KPC-2_ (5’-GCGGCAGAGCCATATGGAGCGCTTCAACGGTCGTATC-3’ and 5’-GCTCCTCGAGGCTCTGCCGCGCAGACTC-3’) and *bla*_CTX-M-65_/*bla*_CTX-M-249_ (5’-AAAGTTCAAACATATGATCAGCAAAAGGGGATGATAAG-3’ and 5’-TGATCTCGAGTTTGAACTTTTGCTTTGCC-3’). The templates for amplification were *E. coli* DH5α-pXM610-2 (*bla*_KPC-157_ and *bla*_CTX-M-65_), *E. coli* DH5α-pXM610-4 (*bla*_CTX-M-249_), and *K. pneumoniae* strain KPCZA02 (*bla*_KPC-2_) (4), respectively. The products were digested with NdeI and XhoI and ligated into pET-28a (Novagen, USA). The ligation mixture was transformed into *E. coli* DH5α. Recombinant clones were selected on MHA plates containing 50 mg/L kanamycin and 1 mg/L ceftazidime. Inserts were verified by PCR and Sanger sequencing.

**Stability assay**

Strain XM610 was streaked onto antibiotic-free MHA plate from frozen stock. After 24h of incubation at 37^o^C, a single colony was randomly selected and re-streaked onto a fresh antibiotic-free MHA plate (passage 1), and incubated under the same conditions. The cultures from each day were preserved at -80^o^C for further analysis. After being serially passaged for 30 days, cultures on days 0, 5, 10, 15, 20, 25 and 30 were selected for antimicrobial susceptibility testing and Illumina sequencing. The stability of *bla*_KPC-157_ and *bla*_CTX-M-249_ were confirmed by both unchanged resistance patterns and sequences.

**Whole-genome sequencing (WGS) and analysis**

Genomic DNA was sequenced on Illumina platforms with PE150 strategy in Novogene Bioinformatics Technology Co., Ltd (Beijing, China). For XM610 and XM991, long-read Nanopore sequencing was also performed in Novogene Bioinformatics Technology Co., Ltd (Beijing, China). In detail, genomic DNA was extracted with the SDS method (5), quantified with NanoDrop™ 2000 (Thermo Scientific, Waltham, MA), and integrity verified by agarose gel electrophoresis. >5 μg DNA per sample was used to construct libraries using the SQK-LSK110 Ligation Sequencing Kit and the PCR-Free EXP-NBD104/114 Native Barcode Expansion Kit, omitting mechanical shearing to enrich long reads. Final libraries were quantitated with a Qubit 4.0 fluorometer and sequenced on an Oxford Nanopore PromethION platform. Reads were assembled by SPAdes (Version 3.15.5) or Unicycler (Version 0.5.1), and annotated by NCBI Prokaryotic Genome Annotation Pipeline (PGAP). Single nucleotide polymorphisms (SNPs), multilocus sequencing typing (MLST), antibiotic resistance genes and plasmid replicons were analyzed by CSI Phylogeny 1.4 (6), MLST 2.0 (7), ResFind 4.6 (8), and PlasmidFinder 2.0 (9), respectively, on CGE online tools platform (https://www.genomicepidemiology.org/services/). Kaptive was used to identify K-locus (KL) (10). Comparison of plasmid sequences and sequence alignment were performed using Easyfig2.2 (11).


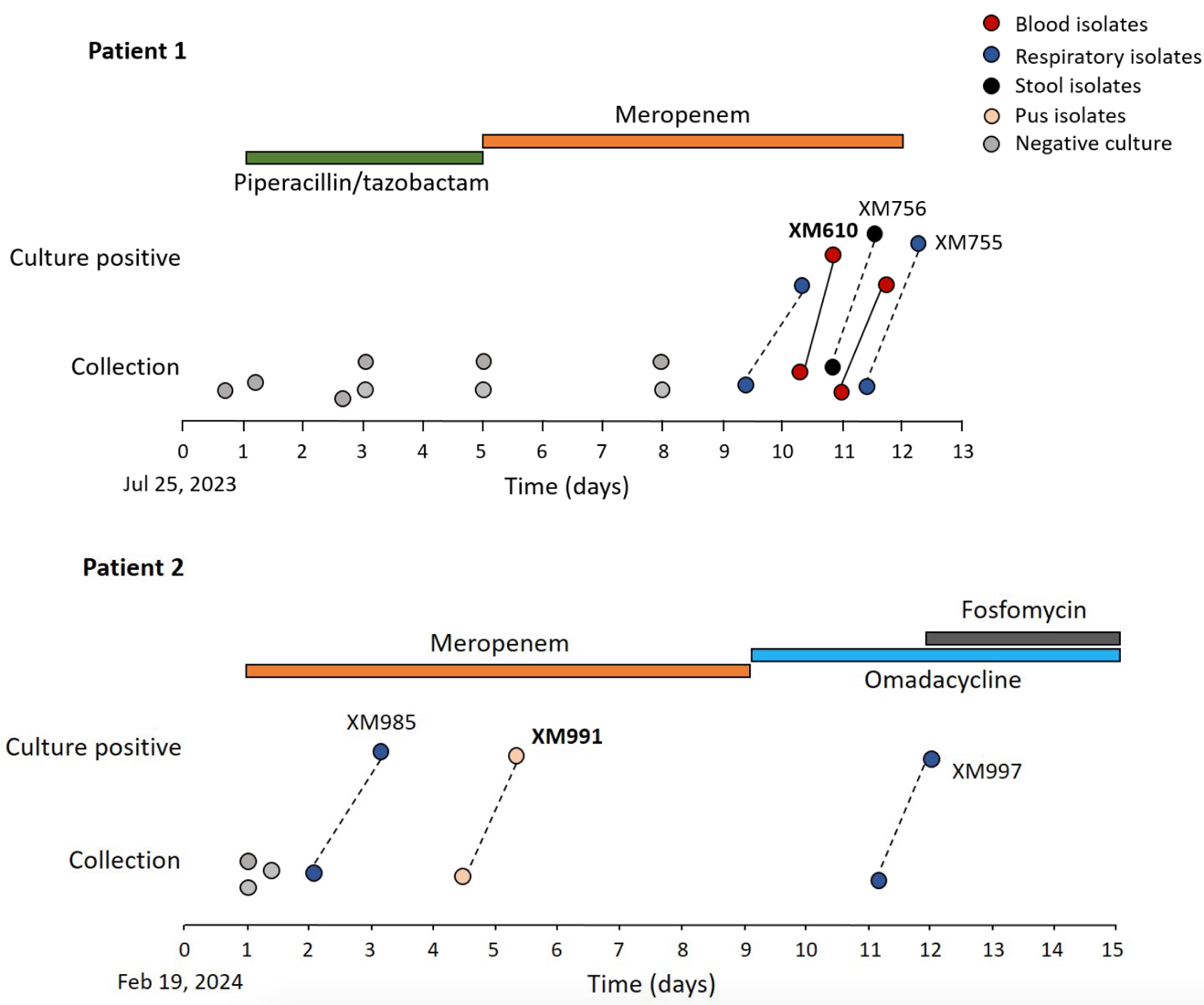


Figure S1. Timeline and antibiotic treatment of the two patients infected with KPC-157 and CTX-M-249 co-harboring *Klebsiella pneumoniae*. The isolates highlighted in bold were subjected to long-read Nanopore sequencing.


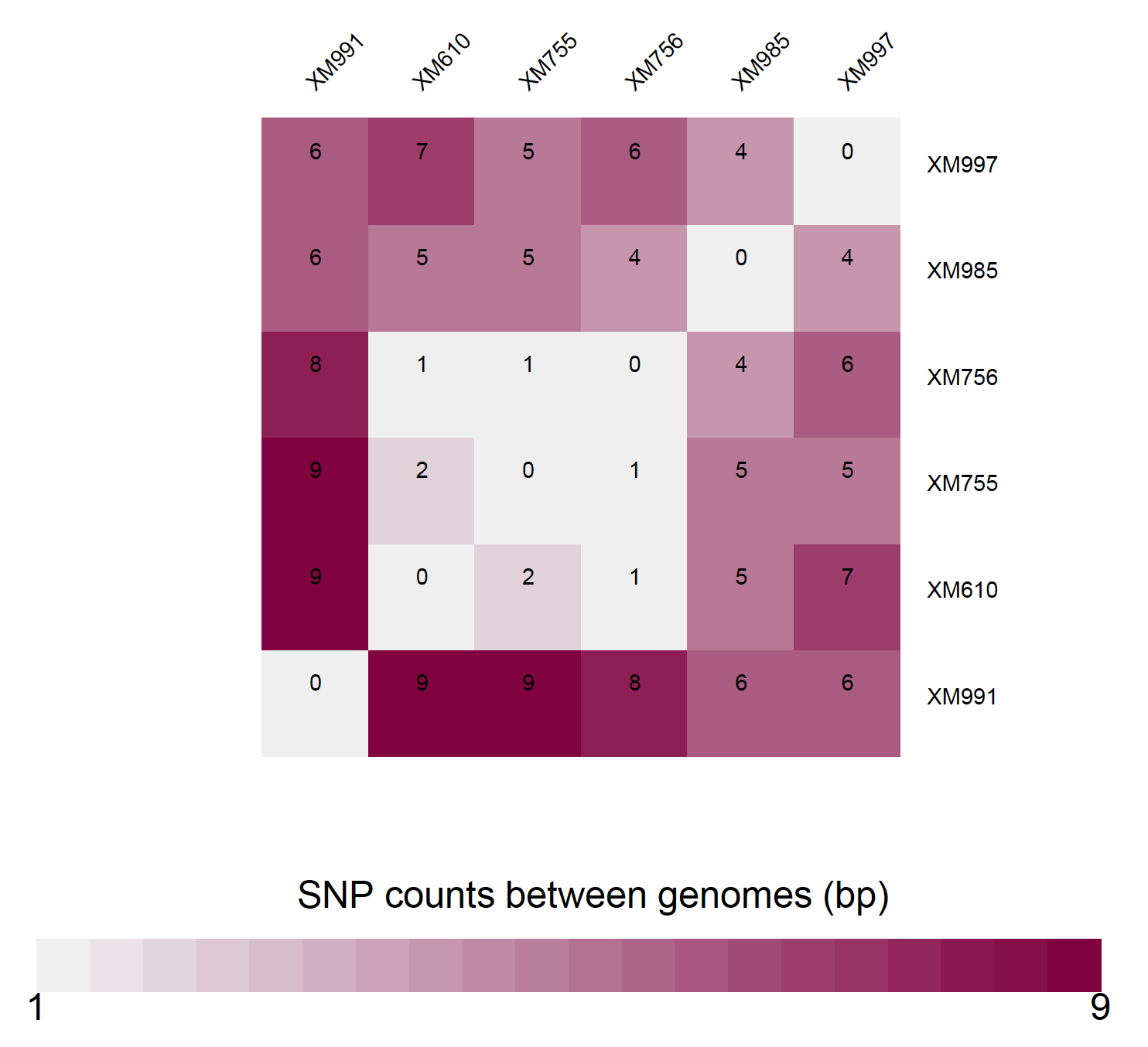


Figure S2. The single-nucleotide polymorphism (SNP) matrix of the six KPC-157 and CTX-M-249 co-harboring *Klebsiella pneumoniae* isolates.

Table S1. Genomic characteristics of *Klebsiella pneumoniae* strains XM610 and XM991

|  | Size (bp) | Replicon types (Identity %) | Resistant genes | Outer membrane proteins |
| --- | --- | --- | --- | --- |
| XM610 |  |  |  |  |
| Chromosome | 5382503 | - | *aadA2b*, *bla*_SHV-182_, *fosA6*, *sul1* | ompK35 p.L63* - Premature stop codon;  ompK36 p.L191* - Premature stop codon;  ompK37 p.K251* - Premature stop codon |
| pXM610-1 | 192256 | IncHI1B (99.15) | None | - |
| pXM610-2 | 141448 | IncFII (81.32) | *rmtB1*, *bla*_KPC-157_, *bla*_CTX-M-65_, *bla*_SHV-12_, *bla*_TEM-1B_, *bla*_LAP-2_, *catA2*, *qnrS1*, *sul2*, *tet(A)*, *dfrA14* | - |
| pXM610-3 | 115212 | IncFIB (99.64) | None | - |
| pXM610-4 | 88638 | IncFII (100);  IncR (99.2) | *bla*_CTX-M-249_ | - |
| pXM610-5 | 11970 | ColRNAI (98.46) | None | - |
|  |  |  |  |  |
| XM991 |  |  |  |  |
| Chromosome | 5386235 | - | *aadA2b*, *bla*_SHV-182_, *fosA6*, *sul1* | ompK35 p.L63* - Premature stop codon;  ompK36 p.L191* - Premature stop codon;  ompK37 p.K251* - Premature stop codon |
| pXM991-1 | 191904 | IncHI1B (99.15) | None | - |
| pXM991-2 | 141448 | IncFII (81.32) | *rmtB1*, *bla*_KPC-157_, *bla*_CTX-M-65_, *bla*_SHV-12_, *bla*_TEM-1B_, *bla*_LAP-2_, *catA2*, *qnrS1*, *sul2*, *tet(A)*, *dfrA14* | - |
| pXM991-3 | 115078 | IncFIB (99.64) | None | - |
| pXM991-4 | 88638 | IncFII (100);  IncR (99.2) | *bla*_CTX-M-249_ | - |
| pXM991-5 | 11970 | ColRNAI (98.46) | None | - |

**References**

1. CLSI. 2025. Performance standards for antimicrobial susceptibility testing. 35th ed. CLSI supplement M100. Wayne PA: Clinical and Laboratory Standards Institute.

2. The European Committee on Antimicrobial Susceptibility Testing. Breakpoint tables for interpretation of MICs and zone diameters. Version 15.0, 2025.

3. Choi KH, Kumar A, Schweizer HP. 2006. A 10-min method for preparation of highly electrocompetent *Pseudomonas aeruginosa* cells: application for DNA fragment transfer between chromosomes and plasmid transformation. J Microbiol Methods 64:391-397. https://doi.org/10.1016/j.mimet.2005.06.001

4. Xu M, Zhao J, Xu L, Yang Q, Xu H, Kong H, Zhou J, Fu Y. 2022. Emergence of transferable ceftazidime-avibactam resistance in KPC-producing *Klebsiella pneumoniae* due to a novel CMY AmpC β-lactamase in China. Clin Microbiol Infect 28:136 e131-136 e136. https://doi.org/10.1016/j.cmi.2021.05.026

5. Lim HJ, Lee EH, Yoon Y, Chua B, Son A. 2016. Portable lysis apparatus for rapid single-step DNA extraction of *Bacillus subtilis*. J Appl Microbiol 120:379-387. https://doi.org/10.1111/jam.13011

6. Kaas RS, Leekitcharoenphon P, Aarestrup FM, Lund O. 2014. Solving the problem of comparing whole bacterial genomes across different sequencing platforms. PLoS One 9:e104984. https://doi.org/10.1371/journal.pone.0104984.

7. Larsen MV, Cosentino S, Rasmussen S, Friis C, Hasman H, Marvig RL, Jelsbak L, Sicheritz-Pontén T, Ussery DW, Aarestrup FM, Lund O. 2012. Multilocus Sequence Typing of Total-Genome-Sequenced Bacteria. J Clin Microbiol 50:1355-61. https://doi.org/10.1128/jcm.06094-11

8. Bortolaia V, Kaas RS, Ruppe E, Roberts MC, Schwarz S, Cattoir V, Philippon A, Allesoe RL, Rebelo AR, Florensa AF, Fagelhauer L, Chakraborty T, Neumann B, Werner G, Bender JK, Stingl K, Nguyen M, Coppens J, Xavier BB, Malhotra-Kumar S, Westh H, Pinholt M, Anjum MF, Duggett NA, Kempf I, Nykäsenoja S, Olkkola S, Wieczorek K, Amaro A, Clemente L, Mossong J, Losch S, Ragimbeau C, Lund O, Aarestrup FM. 2020. ResFinder 4.0 for predictions of phenotypes from genotypes. J Antimicrob Chemother 75:3491-3500. https://doi.org/10.1093/jac/dkaa345

9. Carattoli A, Zankari E, García-Fernández A, Voldby Larsen M, Lund O, Villa L, Møller Aarestrup F, Hasman H. 2014. In Silico Detection and Typing of Plasmids using PlasmidFinder and Plasmid Multilocus Sequence Typing. Antimicrob Agents Chemother 58:3895-903. https://doi.org/10.1128/aac.02412-14

10. Stanton TD, Hetland MAK, Löhr IH, Holt KE, Wyres KL. 2025. Fast and accurate in silico antigen typing with Kaptive 3. Microb Genom 11:001428. https://doi.org/10.1099/mgen.0.001428

11. Sullivan MJ, Petty NK, Beatson SA. 2011. Easyfig: a genome comparison visualizer. Bioinformatics 27:1009-10. https://doi.org/10.1093/bioinformatics/btr039
